# Modulation of the Activity of a Polycomb-Group Response Element in *Drosophila* by a Mutation in the Transcriptional Activator Woc

**DOI:** 10.1534/g3.111.001230

**Published:** 2011-11-01

**Authors:** Amanda Noyes, Catherine Stefaniuk, Yuzhong Cheng, James A. Kennison, Judith A. Kassis

**Affiliations:** Program on Genomics of Differentiation, *Eunice Kennedy Shriver* National Institute of Child Health and Human Development, National Institutes of Health, Bethesda, Maryland 20892

**Keywords:** polycomb repression, polycomb response elements, PRE, *woc*

## Abstract

Polycomb group response elements (PRE) are *cis*-regulatory elements that bind Polycomb group proteins. We are studying a 181-bp PRE from the *Drosophila* *engrailed* gene. This PRE causes pairing-sensitive silencing of mini-*white* in transgenes. Here we show that the 181-bp PRE also represses mini-*white* expression in flies with only one copy of the transgene. To isolate mutations that alter the activity of the 181-bp PRE, we screened for dominant suppressors of PRE-mediated mini-*white* repression. Dominant suppressors of mini-*white* repression were rare; we recovered only nine mutations out of 68,274 progeny screened. Two of the nine mutations isolated are due to the same single amino acid change in the transcriptional activator Woc (without children). Reversion experiments show that these are dominant gain-of-function mutations in *woc*. We suggest that Woc can interfere with the activity of the PRE. Our data have implications for how Polycomb group proteins act to either partially repress or completely silence their target genes.

Polycomb group genes (PcG) encode proteins that mediate transcriptional repression. First identified in *Drosophila* as genes necessary to maintain the silencing of homeotic genes, it is now evident that PcG proteins have many other targets (reviewed in Simon and Kingston 2009). Genome-wide studies show that the PcG repressive mark H3K27me3 is associated with hundreds of genes in single cell types and that targets can be cell-type specific (reviewed in Schwartz and Pirrotta 2008). Although it is evident that PcG proteins can decrease expression levels in addition to completely silencing expression, it is not clear what determines whether a gene will be completely or only partially repressed.

In *Drosophila*, PcG proteins are associated with Polycomb group response elements (PRE), DNA elements that recruit PcG proteins to the DNA (reviewed in Müller and Kassis 2006; Ringrose and Paro 2007). In genome-wide studies, PREs were identified as binding sites for multiple PcG proteins (Schwartz *et al.* 2006; Négre *et al.* 2006; Tolhuis *et al.* 2006). Two functional assays have also been used to identify PREs. In one assay, the PRE is combined in a transgene with regulatory DNA from a gene normally regulated by PcG proteins, where the PRE is required to maintain the “off” transcriptional state (Müller and Bienz 1991; Hagstrom *et al.* 1997). In the other assay, PREs are used to repress expression of the mini-*white* reporter gene in transgenic flies. Because mini-*white* repression is stronger in flies homozygous for the PRE-mini-*white* reporter, this latter assay has been called pairing-sensitive silencing (Kassis 1994).

One of the puzzles of the transgene assays for PREs is that silencing does not occur at every chromosomal insertion site. For example, for the four *engrailed* and *invected* PREs, pairing-sensitive silencing was observed at a frequency of 21–62% of insertion sites (Americo *et al.* 2002; Cunningham *et al.* 2010). PRE activity is regulated by the expression state of the gene it regulates; thus it follows that PRE activity in transgenes is dependent on the activity of regulatory elements that flank the transgene insertion site.

We have been studying a 181-bp *en* DNA fragment that acts as a PRE in several different assays: (1) it represses inappropriate expression in both *en*- and *Ubx*-reporter genes in embryos (Americo *et al.* 2002; Devido *et al.* 2008); (2) PcG proteins are associated with it in tissue culture cells, embryos, larvae, and adults (Strutt and Paro 1997; Négre *et al.* 2006; Oktaba *et al.* 2008); and (3) it acts as a pairing-sensitive silencing element (Kassis 1994). This fragment contains binding sites for the PRE DNA binding proteins Pho, Pho-like, GAGA factor, and Spps (Americo *et al.* 2002; Brown *et al.* 2005; Brown and Kassis 2010). Thus, the 181-bp DNA fragment is clearly a PRE. Therefore, we reasoned that conducting a genetic screen for mutations that alter the activity of this PRE might yield mutations in PcG genes.

We conducted a genetic screen for dominant suppressors of pairing-sensitive silencing by a transgene that contained the 181-bp *en* PRE and mini-*white*. These mutations were rare; we only obtained nine suppressors among 68,274 genomes screened. None of the mutations affected mini-*white* repression of transgenes at all chromosomal insertion sites. This suggests that none of the mutations affects PRE activity directly. Instead, we believe that these mutations affect the expression of genes flanking the transgene insertion site. Consistent with this, two of the dominant suppressors are the same gain-of-function mutation in the gene *without children* (*woc*), which encodes a transcriptional activator. Our data suggest that there is a competition between transcriptional activators and PcG repression and that certain types of activators may be better able to overcome PcG repression.

## Materials and Methods

### Mutagenesis

For EMS mutagenesis, adult males were fed EMS as described (Lewis and Bacher 1968; Kennison 1983), and then discarded 3–4 days following treatment to avoid pre-meiotic clusters of mutations. For the X-ray mutagenesis, males were irradiated with 30–40 Gy at 120 keV using a Faxitron Torrex 2800. The irradiated males were discarded 4–5 days following treatment.

### Sequencing

DNA was isolated from homozygous or hemizygous mutant adults or larvae, and the entire *woc* transcription unit was sequenced.

### Construction of *P[L181PRE]*

The 181-bp *en* PRE was amplified with the primers GCGGAATTCGAGATGGCATGTGGCTCT and GCGGAATTCGCATGCTGGAGCTGTCAG, cut with *Eco*RI, and cloned into *Eco*RI cut, phosphatased EK710, which contains *loxP* sites on both sides of the *Eco*RI site (Kuhn *et al.* 2004). A fragment of DNA containing the 181-bp PRE and flanking *loxP* sites was cut with *Not*I and cloned into *No*tI cut *CaSpeR4*. The resulting clone was sequenced to determine the orientation of the insert.

### Generation and analysis of transgenic lines

*P[L181PRE]* was injected into homozygous *Df(1)w67c23*, *y* embryos using standard techniques. Some lines were generated by *P*-element mobilization by crossing to a strain with the endogenous transposase insertion *P[ry^+^∆2*, *3]99B* (Robertson *et al.* 1988). *P[L]* derivative lines lacking the *en*181bp-PRE were obtained by crossing males with the *P[L181PRE]* insertion to virgin females that carried a constitutively active Cre recombinase transgene (*y^1^w; CyO*, *P[Crew]/Sco*) (Siegal and Hartl 1996). Progeny that contained both *P[L181PRE]* and *CyO*, *P[w+Cre]* were crossed to *Df(1)w67c23*, *y* flies. Two individual *w^+^* male progeny were selected from each insertion line and crossed to the appropriate balancer chromosome. *P[L]* lines were established, and the deletion of the *en*181bp-PRE was confirmed by PCR with primers flanking the *loxP* sites.

### qRT-PCR

Flies of the following genotypes were used: (1) *w^1118^*, (2) *w^1118^; P[181PRE]8-10C*, (3) *w^1118^; woc^D1^*, and (4) *w^1118^;P[181PRE]8-10C; woc^D1^*. Total RNA from 3^rd^ instar larvae, 1-day-old pupae, or adult fly heads was prepared (Lorenz *et al.* 1989) and treated with DNase I before use. qRT-PCR was done with the QuantiTect SYBR Green RT-PCR kit (Qiagen) on the LightCycler 480 real-time PCR system (Roche Applied Sciences) using 0.2 μg total RNA/reaction. The following PCR primers were used: for the *RpL32* reference gene, CGGATCGATATGCTAAGCTGT and CGACGCACTCTGTTGTCG, its amplicon is 67 bp; for *CG30456*, AAAATGCGCAACGATTTCC and AACTTGCCCACCAAATGCT, its amplicon is 95 bp; for *GstS1*, GTCAAGGACAACGATGGTCA and GGTGATGCCTGCGAAGTAG, its amplicon is 72 bp. Reverse transcription was done at 50° for 20 min, followed by incubation at 95° for 15 min to activate the PCR reaction. PCR was for 45 cycles of 94°, 10″, 60° 20″, 72°, 20″. After PCR, the reactions were heated to 95° and then cooled to 40° to analyze the melting temperatures of the PCR products.

## Results

### Dominant modifiers of mini-*white* repression

**Figure 1:**
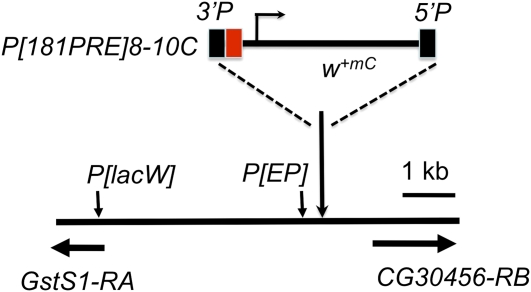
*P[181PRE]8-10C* construct and location in genome. 181-bp *en* PRE (red box) is inserted upstream of the mini-*white* (*m^+mC^*) gene in *P[181PRE]*. *P[181PRE]8-10C* is inserted between *CG30456* and *GstS1*. The 5′ ends of the *GstS1* and *CG30456* transcription units are shown by the horizontal arrows. Vertical arrows indicate the insertion site of the EP element in *P[EP]2185* and the approximate location of a cluster of six *P[lacW]* insertions located within 253 bp of each other near the *GstS1* transcription start site. The insertion site of *P[181PRE]8-10C* is 2R:12989447, 925bp 5′ of the *CG30456-RB* transcription start site and 4512 bp from the 5′ end of *GstS1-RA*.

**Figure 2:**
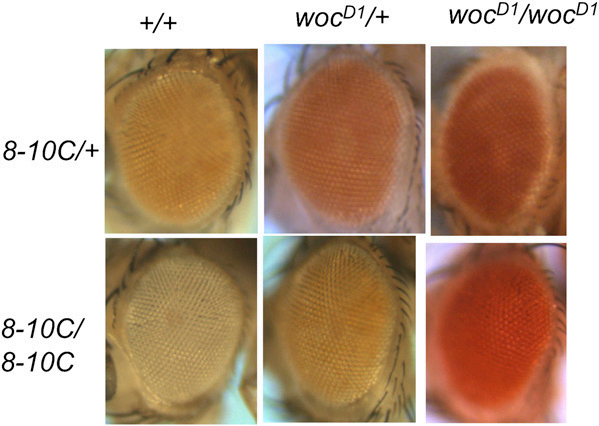
Eye colors of *P[181PRE]8-10C* flies in wildtype and *woc^D1^* mutants. Pictures are of eyes of 1-day-old females. All flies were *w^1118^/w^1118^*, and either homozygous or heterozygous for the *P[181PRE]8-10C* insertion (designated by *8-10C* in the figure) and *woc^D1^* as indicated.

To recover mutations that affect pairing-sensitive silencing, we screened for dominant mutations that suppressed *en*181bp-mediated mini-*white* repression. We used the line *P[181PRE]8-10C*, which contains a P-construct with the 181-bp PRE of *en* DNA cloned into *pCaSpeR* (Construct 8 in Kassis 1994; Figure 1). *pCaSpeR* contains the mini-*white* gene; a truncated version of the *white* gene, which contains a promoter fragment that gives expression in the eye but no eye enhancer. The 181-bp PRE is cloned directly adjacent to the mini-*white* promoter. The *w; P[181PRE]8-10C* homozygotes have white eyes, and *w; P[181PRE]8-10C* heterozygotes have orange eyes (Figure 2). For the mutagenesis, we fed *w; P[181PRE]8-10C* males EMS and crossed to either *w; P[181PRE]8-10C* or *w; P[181PRE]8-10C Sco/CyO* females. We looked for mutations that darkened the eye color of either homozygotes or heterozygotes. We recovered nine mutations; one on the X chromosome, four on chromosome 2, and four on chromosome 3. All but two of the mutations darkened the eye color of both *P[181PRE]8-10C* homozygotes and heterozygotes. These mutations could identify genes involved in repression of mini-*white* transcription, perhaps via the PRE. One second-chromosome mutation darkened the eye color of heterozygotes only, which suggests that it is not involved in mini-*white* repression but might be involved in pigmentation. We did not study this mutation further. The sex-linked mutation only darkens the eye color of *P[181PRE]8-10C* homozygotes. The reason for this is unknown; however, it could mean that the mutation affects the interaction between PREs.

### Two mutations cause the same single amino acid change in the transcriptional activator Woc

We mapped the mutations on chromosome 3 using the markers *ru*, *h*, *th*, *cu*, *sr*, *e^s^*, and *Pr*. Two mutations mapped 2.5 map units distal to *Pr*. We next tried to recover recombinants between these two mutations. We found no recombinants among 448 progeny, suggesting that the two mutations are very close to each other and might be allelic. We tested whether several overlapping deletions for polytene chromosome region 96F–98B (which should include the mutations) caused a darkening of the *P[181PRE]8-10C* heterozygous eye color. As none did, we suspected that both mutations are gain-of-function alleles that produce proteins with altered activities. If so, then a mutation that inactivates the mutant protein should revert the dominant suppression of the *P[181PRE]8-10C* eye color. Therefore, we tried to revert both mutations.

We used both X-rays and EMS to generate revertants. We recovered three X-ray-induced revertants (from 24,758 progeny) and four EMS-induced revertants (from 5500 progeny). The revertants are lethal over deficiencies for the region 96F1–98A5. By crossing to overlapping deficiencies and lethals in the region, we found that all of the revertants are lethal or semilethal mutations in the gene *woc*.

**Figure 3:**
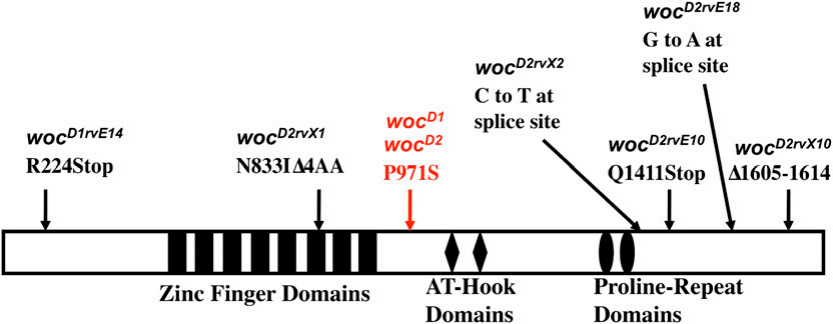
Mutations in the *woc* gene generated in this study. The Woc protein is depicted by the rectangle with identified domains indicated. The location of the dominant suppressors *woc^D1^* and *woc^D2^* is highlighted in red. The mutations present in the revertants are shown. ∆ indicates a deletion of four amino acids in *woc^D2rvX1^*. In *woc^D2rvX10^*, amino acid numbers 1605-1614 are deleted.

Sequencing of the *woc* gene from our original suppressor mutation chromosomes showed that both of these mutations are due to the same single amino acid change in a position evolutionarily conserved throughout the *Drosophila* lineage, as well as in most insects (Figure 3). This amino acid change occurs in a region of the protein with no known domain or function. We named the two original suppressor mutations *woc^D1^* and *woc^D2^* (for *woc^Dominant1^* and *woc^Dominant2^*), and we named the revertant alleles based on the allele reverted and the mutagen used (*i.e.*, *woc^D1rvE8^* was a revertant (rv) generated from *woc^D1^* by EMS (E) mutagenesis). The *wocD1/+* and *wocD2/+* flies have no phenotypic defects. The *woc^D1^* and *woc^D2^* homozygotes survive, are fertile, and also show no phenotypic defects.

We also sequenced the revertants. As expected, all revertants contained the mutation present in *woc^D1^* and *woc^D2^*, as well as an additional lesion in the *woc* transcription unit (Figure 3). With the exception of *woc^D2rvX1^*, all of the mutants were lethal when heterozygous with all other *woc* mutants. The *woc^D2rvX1^*, which contains a four amino acid deletion in the sixth zinc finger, is a hypomorphic allele. The *woc^D2rvX1^* survives poorly in combination with the other hypomorphic *woc* alleles, *woc^rgl^ and woc^468^*. Transheterozygous *woc^D2rvX1^/woc^rgl^* and *woc^D2rvX1^/woc^468^* flies have multiple phenotypic defects, including downturned wings, lack of wing veins, slightly rough eyes, and they are sterile.

### *woc^D^* suppresses the eye color in a position-specific manner

*P[181PRE]8-10C* is inserted in the genome between the genes *GstS1* and *CG30456* (Figure 1). We wanted to know whether *woc^D^* modulates the PRE directly or whether it acts through regulatory DNA flanking the insertion site of *P[181PRE]8-10C*. Importantly, *woc^D^* does not darken the eye color of *w^a^*, a mutation in the *w* gene that reduces the amount of *w* transcript and leads to orange eyes (Pirrotta and Bröckl 1984; Levis *et al.* 1984). This shows that *woc^D^* does not darken eye color indiscriminately. We examined whether the eye colors of flies heterozygous for other mini-*white* containing transgenes inserted near *GstS1* were altered by *woc^D^*. We used a line with a *P[EP]* element inserted about 1.2 kb away from the insertion site of *P[181PRE]8-10C* and six lines with a *P[lacW]* inserted in the promoter region of *GstS1* (Figure 1). The eye colors of *P[lacW]* or *P[EP]/+*; *+/TM6C* were compared with the eye colors of *P[lacW]* or *P[EP]/+*; *woc^D1^/+* flies; no eye color differences were observed. This suggests that the effect of *woc^D^* on the eye color of *P[181PRE]8-10C* flies is dependent on the presence of the PRE in the transgene.

We next examined whether *woc^D^* could alter the eye color of flies with *P[181PRE]* inserted at different chromosomal locations. We used the transgene *P[L181PRE]*, which contains the same 181-PRE as in *P[181PRE]8-10C*. In *P[L181PRE]*, the 181-bp PRE is flanked by *loxP* sites (see below). Because *woc^D^* dominantly alters the eye color of *P[181PRE]8-10C* heterozygotes, we looked at whether *woc^D^* could dominantly alter the eye color of flies heterozygous for *P[L181PRE]* insertions that show mini-*white* repression. For 14 out of 15 *P[L181PRE]* lines tested, *woc^D^* does not alter the eye color. However, in *P[L181PRE]-8A*, the eye color was slightly darker in a *woc^D^* mutant (data not shown). *P[L181PRE]-8A* is inserted just upstream of the PcG-regulated gene *CycA* (at 3L:*11826614*). To determine whether the effect on the eye color of *P[L181PRE]-8A* flies was due to the PRE, we examined the eye color of *P[L]-8A* flies in which the 181-bp PRE had been removed. We found that *woc^D^* had no effect on the eye color of flies that lacked the PRE. This shows that, at least at this chromosomal location, the change in eye color mediated by *woc^D^* is dependent on the PRE. However, as *woc^D^* does not influence the eye color of most *P[L181PRE]* lines, we believe that *woc^D^* is not working on the PRE directly but on sequences flanking the *P[181PRE]* insertion sites.

### *woc^D^* increases the levels of *GstS1* RNA in adult heads

**Figure 4:**
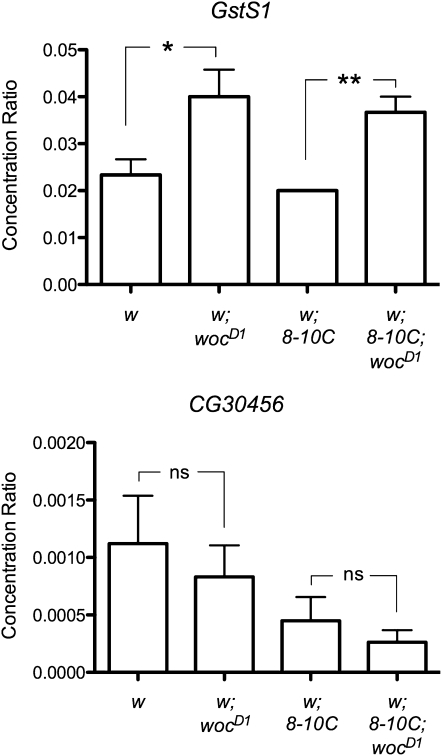
The *woc^D1^* mutation increases the amount of *GstS1* transcript in adult heads. Graph shows the concentration ratio of *GstS1* and *CG30456* RNA to *RpL32* RNA in fly heads (by qRT-PCR). The symbol *8-10C* refers to *P[181PRE]8-10C*. Comparisons were made between wild type and *woc^D1^* homozygotes, in flies homozygous for *P[181PRE]8-10C* or lacking it entirely. *woc^D1^* increased the amount of *GstS1* RNA about 2-fold regardless of whether *P[181PRE]8-10C* was present. *woc^D1^* has no significant effect on *CG30456* RNA levels. Results of three independent experiments are combined and SEM is shown. *P ≤ 0.05; **P ≤ 0.01; ns = not significant as determined by unpaired *t*-tests.

We examined whether the levels of *GstS1* and *CG30456* transcripts were altered in *woc^D1^* mutants, both in the presence and in the absence of the *P[181PRE]8-10C* insertion. We examined RNA levels at three developmental stages: 3^rd^ instar larvae, 1-day-old pupae, and adult heads. We saw no significant differences in the expression levels of *GstS1* and *CG30456* between *woc^D1^* and wild-type 3^rd^ instar larvae or one-day old pupae (data not shown). However, we saw a twofold increase in the level of *GstS1*-RNA in the adult heads of homozygous *woc^D1^* mutants compared to wild-type (Figure 4). Flies with the *P[181PRE]8-10C* insert had about a twofold decrease in the expression levels of *CG30456* at all developmental stages, suggesting that the insertion interferes with the transcription of *CG30456* (Figure 4 and data not shown). However, *woc^D1^* had no significant effect on the transcription level of *CG30456* at any developmental stage either in wild-type or in *P[181PRE]8-10C* animals (Figure 4 and data not shown). We also examined *GstS1* and *CG30456* transcript levels in eye-antennal disks from third instar larvae of wild-type and *woc^D1^* mutants with *P[181PRE]8-10C* and saw no significant differences (data not shown). Finally, we tested whether *woc^D^* altered the transcription level of *CycA* in adult heads. We saw no significant differences in *CycA* levels between wild-type and *woc^D1^*; *P[181PRE]* heads (data not shown).

### Other dominant suppressors do not affect the expression level of *GstS1* or *CG30456*

We tested whether four of our other dominant suppressors of pairing-sensitive silencing of *P[181PRE]8-10C* effect the expression levels of *GstS1* or *CG30465* in fly heads by qRT-PCR; we saw no effect on transcript levels of either gene (data not shown). Thus, a change in transcription level of a *GstS1* is not required for suppression of the pairing-sensitive silencing of *P[181PRE]8-10C*. Finally, we examined the effects of three the other suppressor mutants on the eye color of *P[L181PRE]* at multiple insertion sites. Like *woc^D^*, none of the other suppressor mutations altered the eye color of *P[L181PRE]* at the insertion sites.

### A single unpaired copy of the PRE reduces the eye color of mini-*white* transformants

**Figure 5:**
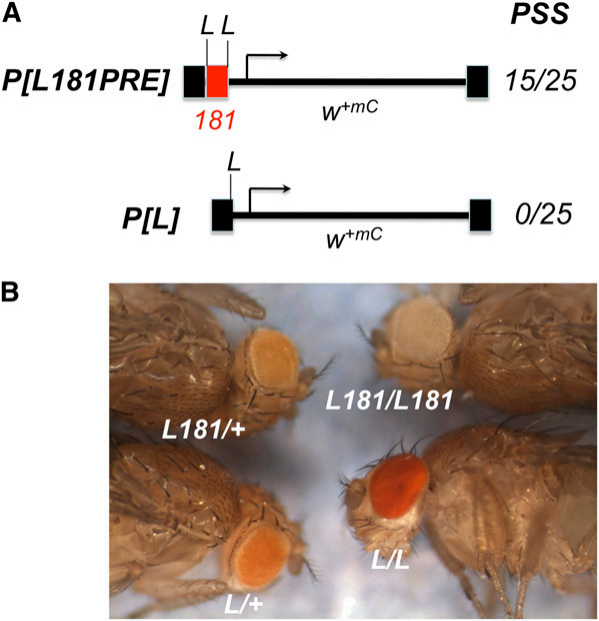
*P[L181PRE]* construct and eye colors. (A) *P[L181PRE]* is identical *to P[181PRE]* except the 181-bp *en* PRE is flanked by *loxP* sites (L). PSS is the number of lines with pairing-sensitive silencing/the total number of viable lines obtained. (B) Eyes from 2-day-old males of lines *P[L181PRE]C2A* (*L181*) and *P[L]C2A* (*L*) are shown. Note that the *P[L]*/+ eyes are darker than the *P[L181PRE]*/+ eyes.

We flanked the 181-bp PRE by *loxP* sites and cloned it upstream of the mini-*white* reporter in *pCaSpeR* (*P[L181PRE]*), in the same orientation and position as in the construct *P[181PRE]* (Figure 5). We recovered 32 lines with insertions of *P[L181PRE]*. Of the 25 insertion lines that were homozygous viable, 15 exhibited pairing-sensitive silencing (60%). All lines were treated with Cre recombinase to excise the 181-bp PRE, yielding *P[L]*. None of the lines without the PRE showed pairing-sensitive silencing. In 7 of the 15 pairing-sensitive lines, the eye color of heterozygous flies became darker upon removal of the PRE, showing that some repression of mini-*white* expression occurred even in the heterozygotes (Figure 5). In contrast, in the lines that did not show pairing-sensitive silencing, the eye colors of flies heterozygous or homozygous for *P[L181PRE]* did not change after removal of the PRE, with one exception. In line *P[L181PRE]11A*, the eye color was slightly lighter after removal of the PRE, suggesting that this element was acting as a slight activator of mini-*white* expression at this location. This result is consistent with earlier evidence that showed that the 181-bp fragment, in the context of a reporter gene driven by the *en* promoter, can act as either an activator or repressor of gene expression depending on the context (Devido *et al.* 2008). This activation activity was weak and only occurred in 1 line out of 32. This shows that, in the mini-*white* assay, PRE-mediated repression is the usual situation.

We determined the chromosomal insertion site for 26 of the *P[L181PRE]* lines (9 lines were lost prior to this part of the analysis) and examined whether the insertion occurred in or near a transcription unit (Table 1). We also examined whether the nearby gene is transcribed in the eye. We note that lines with pairing-sensitive silencing were just as likely to be inserted in or near genes transcribed in the eye as lines without it. Thus, insertion near a gene that is transcribed does not interfere with pairing-sensitive silencing.

**Table 1:** *P[L181PRE]* insertions

Line Name	Location*^a^*	Gene*^b^*	Distance*^c^*	Transcript Level in Eye*^d^*
Lines that show pairing-sensitive silencing				
C2A	3R:9882721	*Foxo*	+31bp	48
C5A	X:9581402	*Nej*	+303bp	NID
C6A	X:18398636	*Wnt5*	+800bp	18
C11B	3R:9487247	*B52*	+255bp	NID
1A	X:19498690	*CG14207*	−501bp	300
1B	2L:11106592	*Reps*	+60bp	27
12A	3R:21867498	*Gro*	−66bp	197
14A	3R:7590188	*Lk6*	−9bp	4362
24A	2R:2051740	*Lbk*	+150bp	138
15A	3L:19922217	*RhoGDI*	+310bp	4481
23A	2L:10414068	*Klp31E*	−34bp	98
8A	3L:11826614	*CycA*	−81bp	NID
Lines that do not show pairing-sensitive silencing				
C1	2R:3623151	*CG18812*	−27bp	728
2	3L:12074850	*Sema-5c*	+77bp	47
C1-12A	3L:13932279	*CG32137*	−73bp	146
13A	X:14983581	*rab3-GEF*	−134bp	173
16A	2L:2753118	*CG9894*	+319bp	2886
17A	2R:13435831	*MESR4*	+502bp	41
28A	2R:15556892	*Hrg*	−325bp	383
10	2L:3477289	*Thor*	−1145bp	1016
21B	2R:8475807	*Sin3A*	+793bp	NID

Three lethal lines are included in this table: 8A, 10, and 21B. The eye color of line 8A heterozygous flies became lighter upon excision of the PRE; thus, we consider that this line undergoes mini-*white* repression by the PRE and classify it as having pairing-sensitive silencing. The eye colors of lines 10 and 21B did not change upon excision of the PRE and are classified as lines that do not show pairing-sensitive silencing. NID, no informative data.

a Insertion site of the *P[L181PRE]*, genome version R5.39.

b Nearest gene (http://flybase.org; Tweedie *et al.* 2009).

c Distance to the nearest transcription start site. Positive numbers indicate it is within the transcription unit. Negative numbers indicate it is upstream of the transcription unit.

d Transcript level in the adult eye. Data are taken from the FlyAtlas Organ/Tissue Expression as listed on Flybase (http://flybase.org). Low (10–99.9), moderate (100–499.9), high (500–999.9), and very high expression (1000–25,000).

## Discussion

To gain insight into the mechanism of pairing-sensitive silencing mediated by the 181-bp *en* PRE, we conducted a genetic screen for dominant mutations that affected mini-*white* repression by that element. Notably, we obtained only nine mutations from screening 68,274 progeny. This low frequency of mutation recovery suggests that loss-of-function alleles were not obtained in our screen. If the loss of one copy of a gene could affect mini-*white* repression, these mutations would have been much more frequent. Our data suggest that mini-*white* repression by the 181-bp PRE is not dependent on genes that are dosage sensitive.

The classical Polycomb group mutant phenotype is the presence of sex comb teeth on the second and third legs, caused by derepression of the *Sex combs reduced* (*Scr*) HOX gene (reviewed in Kennison 1995). *Scr* repression is sensitive to the dose of some PcG genes, as flies with only one wild-type copy have sex comb teeth on the second and third legs. In contrast, mini-*white* repression via the *en* 181bp PRE is not sensitive to a reduction in dosage of the PcG genes (Kassis 1994 and unpublished data). The 181-bp PRE is known to bind PcG proteins, and a binding site for the DNA-binding PcG group protein Pho is required for 181-bp–mediated mini-*white* repression (Brown *et al.* 1998). Thus, we believe that PcG proteins mediate *en* PRE mini-*white* repression, but that this target of PcG proteins is not dosage sensitive.

### *woc^D1^* and *woc^D2^* are gain-of-function mutations

Heterozygosity for either *woc^D1^* or *woc^D2^* darkens the eye colors of *P[181PRE]8-10C* flies, whereas heterozygosity of *woc* deletions does not. This shows that *woc^D1^* and *woc^D2^* have acquired new activities and are gain-of-function alleles. Our results also show that wild-type Woc protein competes with WocD protein. This is suggested by the observation that *P[181PRE]8-10C/P[181PRE]8-10C; woc^D^/woc^−^* flies have a darker eye color than *P[181PRE]8-10C/P[181PRE]8-10C; woc^D^/^+^* flies. Finally, the observation that the activity of WocD is abrogated by mutations that inactivate the Woc protein shows that the *woc^D^* mutation alters the activity of the protein.

### A model for WocD

The *woc* gene encodes a zinc-finger transcription factor implicated in transcriptional activation (Wismar *et al.* 2000; Raffa *et al.* 2005) that acts, at least in part, through an association with HP1c (Font-Burgada *et al.* 2008; Able *et al.* 2009). There are five HP1 isoforms in *Drosophila*. Of these, HP1a is the best studied and is associated with heterochromatic DNA. In contrast, HP1c is excluded from centromeric heterochromatin and is associated with euchromatin.

As stated above *woc^D^* suppresses mini-*white* expression from *P[181PRE]* in a position-dependent manner. Therefore, we wanted to know whether Woc binds to the genomic regions near the *P[181PRE]* insertion sites it regulates. We were not able to obtain Woc antisera, and there is no published data showing where Woc binds in the genome. However, the *Drosophila* ModENCODE project has mapped the binding sites of HP1c in four different cell culture lines by chromatin immunoprecipitation followed by hybridization to tiling arrays (Kharchendo *et al.* 2010). Because Woc is often associated with HP1c, we examined whether there was a correlation between HP1c binding and the suppressor activity of *woc^D^*. HP1c is bound in a cell-type–specific manner. There is no HP1c associated with the region between *GstS1* and *CG30456* in S2, Kc167, or BG3 cells; however, HP1c is bound to this region in clone 8 cells. HP1c is not associated with *CycA* in any cell type. There is no data on HP1c localization in the pigment cells in the eye, so we cannot make any conclusion about whether WocD acts via HP1c in suppressing the PRE activity of *P[181PRE]8-10C*.

**Figure 6:**
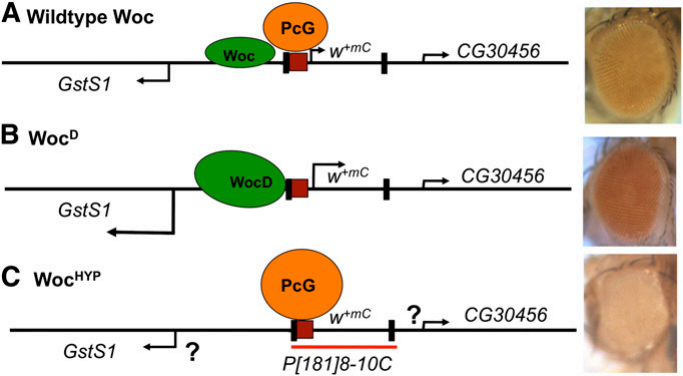
Interplay between Woc and PcG activity determines the eye colors of *P[181PRE]8-10C* flies. Genomic DNA around the *P[181PRE]8-10C* insertion site is denoted by the black line. The *CG30456*, *GstS1*, and *w^+mC^* promoters are designated by arrows pointing in the direction of transcription, with the height of the arrow indicating the relative level of transcription. The P-element ends (black rectangles), the 181-bp *en* PRE (red box), and the extent of the *P[181PRE]* transgene (red line at bottom) are shown. Green ovals indicate Woc activity, with WocD a bigger shape to indicate a higher activity. (Note that we have no evidence that Woc binds directly to this location.) PcG proteins are represented by orange ovals, with the level of repression indicated by the size of the oval. Eyes from flies of the genotype (A) *w^1118^; P[181PRE]8-10C/+* (B) *w^1118^; P[181PRE]8-10C/+; woc^D1^/+* (C) *w^1118^; P[181PRE]8-10C/+; woc^D2rvX1^/woc^rgl^* are shown on the right. Woc^HYP^ indicates a hypomorphic allelic combination. We were not able to obtain enough *woc^HYP^* adults to perform qRT-PCR, so we do not know the level of *CG30456* and *GstS1* RNA in these flies. This uncertainty is indicated by the question mark next to the transcription arrows in (C).

How does *woc^D^* affect the eye color of *P[181PRE]8-10C*? We suggest that the eye colors of *P[181PRE]8-10C* flies result from a competition of transcriptional repression (caused by the PRE) and transcriptional activation of *GstS1* (Figure 6). In the wild-type case, we suggest there is a competition between transcriptional activation of mini-*white* by flanking regulatory DNA and transcriptional repression mediated by the PRE, leading to an intermediate eye color. One prediction of this model is that if Woc levels are decreased, the PRE upstream of mini-*white* should be able to work more strongly, and the eye color should be lighter. Consistent with this hypothesis, the eye color of *P[181PRE]8-10C/+*; *woc^rgl^/woc^D2X1^* flies is white (Figure 6). We suggest that the PRE in line *P[181PRE]8-10C* modulates the levels of mini-*white* expression in part through a competition with flanking activators.

### What determines PRE activity?

PREs have been studied for many years as silencers of homeotic gene expression in *Drosophila*. Recent genome-wide studies showed that PREs may play an important role in regulating gene expression levels as well. What determines whether a PRE will completely silence a gene or only decrease its expression levels? Our data suggests two things. First, the number of PREs is important. This is evident from the fact that flies homozygous for PRE-mini-*white* constructs, which have two PREs, repress mini-*white* to a much higher level than flies heterozygous. We also note that increasing the number of PREs in *cis*, by duplicating a P-construct with PREs, also causes an increase in mini-*white* repression (Kassis 1994). Second, changes in the chromatin environment, here caused by a gain-of-function mutation in the transcriptional activator Woc, can inactivate a PRE. The dependence of PRE activity in transgenes on chromosomal environment has long been recognized and is dramatically demonstrated in a recent report showing the effect of chromosome environment on the activities of the *Abd-B Fab-7* PRE and the *vg* PRE (Okulski *et al.* 2011).

The relationship between transcriptional activation and PRE function is not simple. Addition of an enhancer containing three binding sites for the eye enhancer-activator protein Glass (GBS) to *pCaSpeR* darkens the eye color of transformants that contain an *en* PRE (Americo *et al.* 2002). If the increased transcription driven by the GBS enhancer interferes with PRE activity, one would expect to see a decrease in the number of lines with pairing-sensitive silencing in this vector. However, this did not occur. Thus, increasing the transcription of mini-*white* itself does not alter PRE activity. In addition, we found that insertion of *P[L181PRE]* into or next to genes expressed in the eye did not prevent pairing-sensitive silencing from occurring (Table 1). Therefore, we propose that it is not transcriptional activation but the actual activators present that determine whether a PRE is active or not. It has previously been suggested that PREs are general silencer elements that could act on any enhancer (Sengupta *et al.* 2004). The basis for this conclusion was that the *Ubx* PRE could act as silencers of three enhancers in reporter genes [two *vestigal* (*vg*) and one *decapentaplegic* (*dpp*) enhancer]. At that time, neither *vg* nor *dpp* was thought to be regulated by PcG proteins. However, since then, a *vg* PRE and a *dpp* PRE have been identified (Lee *et al.* 2005; Hauenschild *et al.* 2008; Okulski *et al.* 2011). We suggest that PREs may not be able to silence all enhancers, and in some chromosomal locations, they cannot act. It was recently reported that a human tissue–specific enhancer functions in erythroid cells by evicting PcG proteins (Vernimmen *et al.* 2011). Enhancers with this activity may also be present in *Drosophila*.
